# The Patient, the Provider, and the TikTok Creator: Qualitative Analysis of the Content and Quality of Videos on Prenatal Genetic Screening

**DOI:** 10.1111/jmwh.70121

**Published:** 2026-04-13

**Authors:** Erin P. Johnson, Naomi O. Riches, Stephanie Arceneaux, Emma Murdock, Caitlin Quade, Susanna R. Cohen, Robin E. Jensen

**Affiliations:** ^1^ Department of Obstetrics and Gynecology University of Utah Salt Lake City Utah; ^2^ Department of Communication University of Utah Salt Lake City Utah

**Keywords:** health education, noninvasive prenatal testing, social media

## Abstract

**Introduction:**

Although providers may view the use of the noninvasive prenatal testing (NIPT) screen as an opportunity for patients to learn more about potential chromosomal variants of a fetus, research suggests that patients may view the genetic screening test primarily as an opportunity to learn about their fetus's sex chromosomes and may not understand the implications of a screening test, as compared with a diagnostic test. This study critically evaluates the informational quality of TikTok content related to NIPT, with a specific focus on the use and conflation of sex and gender terminology.

**Methods:**

A total of 83 TikTok videos met our inclusion criteria. Two team members systematically coded the videos using a modified DISCERN instrument and content‐specific codes to assess reliability and informational quality across 3 content creator categories: patients/nonexperts, health care professionals, and organizations. Many codes focused specifically on the use of gendered language in the videos.

**Results:**

Patient‐generated content exhibited the highest engagement metrics but frequently misrepresented NIPT as a confirmatory test and conflated sex with gender. Health care provider and organizational videos demonstrated higher informational reliability but were not immune to deterministic language around sex and gender and promotional framing.

**Discussion:**

The findings underscore the influence of social media as a health information source and reveal significant gaps in public understanding of NIPT, particularly regarding its screening nature and limitations. Additionally, these results highlight the need for clinical protocols that address patient misconceptions and suggest that health care professionals should actively engage with digital platforms to familiarize themselves with what patients are seeing and hearing online. Recommendations include avoiding gendered mirroring, asking questions about patient knowledge, and leveraging social media for public health education.

## INTRODUCTION

Advancements in genetic testing have changed the landscape for people who are pregnant and seeking to understand the likelihood that their fetus has a chromosomal or genetic condition. Although these opportunities have historically been offered to individuals “at risk,” including those with a history of genetic or chromosomal conditions, the American College of Obstetricians and Gynecologists (ACOG) now recommends that all individuals who are pregnant be offered prenatal genetic testing as part of their routine care,^1^ regardless of maternal age, disease, health history, or risk status. Traditional invasive prenatal diagnostic tests, such as amniocentesis and chorionic villus sampling, yield the most accurate diagnosis but carry a risk of miscarriage^2^ and are done later in pregnancy. Providers, therefore, typically offer a less invasive first‐line screening test, such as cell‐free DNA (cfDNA) screening. Although cfDNA (also known as noninvasive prenatal testing [NIPT]) is the most accurate among noninvasive screening tests, it still has a low false‐positive rate and provides results as risk estimates, not diagnostic information. Risk and probability are notoriously difficult for patients to understand,^3^ and patients may not comprehend the need for confirmatory testing. As a result, it is important for patients to engage in shared‐decision‐making with their providers to counter any inaccurate information they may have been exposed to.

**QUICK POINTS**

✦ Forty‐two percent of videos conflated sex and gender in chromosomal screening contexts.
✦ Patient videos had the highest engagement but the lowest DISCERN reliability scores.
✦ Noninvasive prenatal testing was frequently misrepresented as confirmatory rather than screening.
✦ Providers should attempt to counter potential inaccuracies patients see online, particularly with regard to sex determination and differences between sex and gender.

### NIPT and the Sex Chromosomes

Although providers may view the use of the NIPT screen as an opportunity for patients to learn more about potential fetal chromosomal variants, research suggests that patients may view the genetic screening test primarily as an opportunity to learn about their fetus's sex chromosomes.^4^ A common question that perinatal providers hear from patients and families is, “When can I find out if my baby is a boy or a girl?”^5^ With NIPT now being offered by primary perinatal providers (eg, obstetricians, midwives) rather than genetic counselors, the provider is increasingly responsible for administering sex prediction results to patients based on a chromosomal result, and many perinatal providers may lack the knowledge or training needed to discuss prenatal fetal sex prediction with parents.^5^

Sex assigned at birth and a fetus's gender are traditionally conflated, particularly at the sociocultural level, contributing to a binary concept for both sex and gender.^6^, ^7^, ^8^ This can result in a range of problems related to, for instance, inadequate care if assumptions about sex and gender overlap with undesired clinical interventions (such as circumcision or surgical intervention for children born with androgen insensitivity syndrome) or even a compromise of health care at a broader level if sex and gender are conflated as demographics in institutional data.^9^ There are entire fields of applied and theoretical study—most notably gender studies, the sociology of gender, and developmental psychology—dedicated to exploring the idea that *gender* is: (1) a social construct, (2) not binary, (3) not determined by biological sex, and (4) potentially fluid. Even chromosomal sex, typically perceived as binary (XX vs XY), has variations such as XXY, XXX, and others, many of which can be screened for using NIPT technology. Variations on biological sex can occur at multiple levels: gonadal sex (testes vs ovaries), hormonal sex (hormones that trigger the development of fallopian tubes, ovaries, or vas deferens, etc), and, by the fourth month of fetal development, formation of genital sex (penis, vulva). The result is a diversity of intersex manifestations beyond the binary of biological sex.^10^ Care providers must combine nuance and accuracy in their explanation of this topic, particularly if they need to counter patient misconceptions around sex diagnosis or gender determination from prenatal screening.

### Health Education

Informal methods for individuals to gain health knowledge have always existed, although the most up‐to‐date and medically accurate information has often been assumed to come from a top‐down approach via trained professionals. This approach traditionally designates patients as relatively passive consumers of health information. However, the health education landscape is rapidly changing. The World Health Organization takes a broader view of what health education entails in its *Health Promotion Glossary of Terms* (2021). Health education, a central component of informed consent, not only provides information but also supports and empowers patients to make health decisions that are right for them and their families.^11^

Given the vast amount of information covered in prenatal clinical visits, the importance and benefit of prenatal screening education accessible outside the clinical setting are clear. Providers acknowledge that short appointment times limit the amount and type of quality information they can provide patients.^12^, ^13^ The limited time available during each prenatal appointment is a notable barrier to shared‐decision‐making about prenatal genetic screening.^14^ Additionally, NIPT is complex, and understanding is heavily dependent on an individual's level of health literacy and their prior experience with pregnancy.

Since the widespread adoption of the internet in the late 20th century, people have been turning to the internet to access health information.^15^, ^16^ In recent years, social media platforms have experienced tremendous growth and are increasingly used for health information, particularly among young people.^17^, ^18^ TikTok, which has been the fastest‐growing social media platform among US‐based users in recent years,^19^ is used by 62% of adults aged 18 to 29 years. Notably, those who identify as women use the site at much higher rates than men (40% vs 25%).^19^

Some people are turning to TikTok for health‐related information to get advice from others with a similar disease or condition, secure social support, and gain more personalized insights into a diagnosis or health experience.^18^ One study found that TikTok users gathered health information equally from both health professionals and TikTok but were more likely to act on health information from health professionals.^18^ The democratization of social media means that the public may consider health education from a health professional equally alongside personal experiences they encounter online.

### Study Aim

With NIPT's increased accessibility and affordability, more people may be looking to learn about genetic screening by turning to the internet and social media. In doing so, they face the difficult task of navigating potentially inaccurate and incomplete information because quality assurance is limited in this context. Health care providers need to know what information their patients are exposed to in order to better serve them. In many cases, this involves directly combating inaccurate information and biased content. The aim of this study was to understand the content and quality of information about prenatal genetic screening on the social media platform TikTok, with a secondary aim of describing and contextualizing the use of gender and sex terminology.

## METHODS

This qualitative analysis focuses on the social media platform TikTok. Videos were selected using the Clockworks TikTok scraper tool^20^ (maintained online by Apify), designed to extract data from TikTok videos, hashtags, and users. Data scraped from the tool provided a static record of the top‐viewed TikTok videos on the download date of January 7, 2025. The hashtags #nipt, #prenatalscreening, and #cfDNA were used to identify the top 100 videos per hashtag.

Exclusion criteria for videos included: duplicate TikTok videos, videos longer than 10 minutes, videos clearly targeting an academic audience (eg, class lecture or conference presentation), videos unrelated to prenatal genetic screening, and non–English‐language videos.

Data from video content were assessed in a 2‐tier process: the first tier focused on coding video elements, including the use of the DISCERN tool, and the second tier focused on extended descriptive notes, quotes, and image capture. The first tier included extracting data into a secure data capture form by 2 research team members (E.M. and S.A.). Reliability across coders was established through double coding of the first 10 videos. Five videos were double coded and then reviewed by the full team (E.P.J., R.E.J., N.O.R., C.Q., S.A., S.R.C., and E.M.) to discuss discrepancies and reach a consensus. A second set of 5 videos was then double coded, with discrepancies again reviewed by the full team. Following the initial reliability training, when coders reached 85% congruence, videos were coded by a single individual, and questions were brought to weekly team meetings.

A complete list of data elements included is provided in Supporting Information: Appendix S1. Some data elements required the coders to conduct an online search, such as whether the content creator represented an organization or an individual. To gauge engagement, we examined the number of views, likes, shares, and comments as documented in the Clockworks data scrape. Additionally, the DISCERN instrument for information quality and accuracy^21^ was used to evaluate the reliability of the information in the videos. DISCERN is a validated instrument (Cronbach's α, 0.74‐0.88, indicating high internal consistency) and was chosen based on previously published TikTok video analyses.^22^, ^23^, ^24^, ^25^, ^26^ We used the Reliability and Quality of Information scales, which include 15 questions and a final overall quality rating question. DISCERN was developed for written health material and uses a 5‐point Likert scale anchored by “No,” “Partially,” and “Yes” to guide users in scoring. We simplified the scale to a 3‐point scale, removing the unlabeled items so that “No” corresponded to one, “Partially” to 2, and “Yes” to 3. This simplification improved usability for audiovisual material and reliability across coders. For an overall quality rating, we used a methodology similar to that of Kim and Kim, in which videos were classified into 5 quality categories.^27^ DISCERN scores were totaled to a range of 15 to 45 points, which were then categorized as excellent (40‐45), good (34‐39), average (28‐33), poor (22‐27), and very poor (<22). The last question on overall impression was not included in the final DISCERN score.

A second coding tier was completed on any video that included: (1) Lesbian, gay, bisexual, transgender, queer (or questioning), intersex, asexual, and more–inclusive imagery or language; (2) gender or sex terms when discussing prenatal genetic screening; (3) mention of sex chromosomes; or (4) mention of an individual's prenatal genetic screening results. The second‐tier coding included capturing screenshots and timestamps of each occurrence of the listed terms or images and a summative qualitative content analysis of each video.^28^ The content analysis guided the codes generated, and each video was coded by 2 members of the research team (E.M. and S.A.), with discrepancies resolved through discussion with the team. If a new code was created, it was applied to each video. Inductive content analysis of codes was done as a team (E.P.J., R.E.J., E.M., S.A., and N.O.R.).^29^

Our inductive content analysis approach was informed by the constant comparative method described by Vears and Gillam.^30^ Coders identified meaning units, such as spoken phrases, captions, or visuals, relevant to how prenatal testing was framed and then assigned and refined codes through iterative comparison across videos. New codes were discussed in weekly meetings and retrospectively applied to previously reviewed videos to maintain consistency. Screenshots and timestamps were captured within Research Electronic Data Capture (REDCap) for each instance of relevant imagery or language to ensure traceability of interpretations. Content was summarized into categories.

We aimed to establish trustworthiness in this qualitative research by abiding by the framework established by Lincoln and Guba^31^ and adapted by Morse ^32^ to use the terminology of mainstream social science. Some criteria do not apply because of this being non–human subject research. We address generalizability by applying multiple hashtag terms and downloading all videos on a single day to ensure a snapshot in time for all video statistics. We address reliability by reviewing analyses and conclusions prepared by team members who were not involved in the coding and analysis. Finally, we address validity by documenting the research team's decisions on how specific audio or visual content should be coded throughout the coding process and by revisiting previously coded videos in light of any documented updates.

Our author team comprises one certified nurse‐midwife, one gender studies specialist, one public health researcher, 2 qualitative researchers in perinatal care and decision science, and 2 research assistants without advanced degrees. Our position as well‐educated academics may influence the interpretation of posted videos, but our team provides a perspective that is both age and racially diverse, with gender identity that aligns with our cohort.

The University of Utah institutional review board classified this project as non–human subject research.

## RESULTS

Presentation of these results and organization of the article were guided by the Standards for Reporting Qualitative Research checklist for qualitative studies (see Supporting Information: Appendix S2).^33^

**Figure 1:**
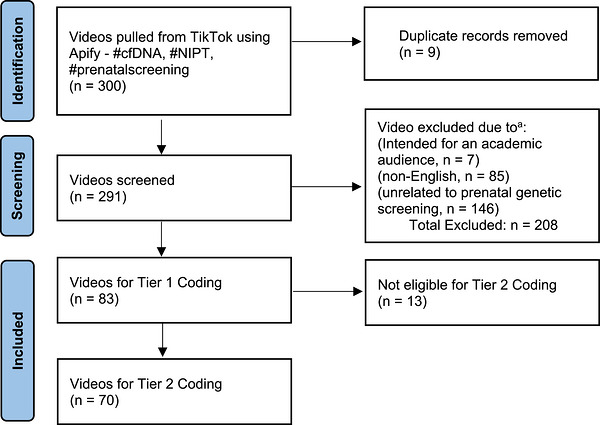
Flowchart for Video Inclusion and Exclusion ^a^Some videos met multiple exclusion criteria.

From the initial 300 TikTok videos screened, 83 were included for tier 1 coding and 70 for tier 2 coding (Figure 1). Exclusions were because of duplication (n = 9), non‐English language (n = 85), unrelated content (eg, DNA filters for motorcycles; n = 146), and academic/clinical audience targeting (n = 7). Some videos met multiple exclusion criteria.

Most videos (83%) were posted by individuals (patients or medical providers), whereas 17% were posted by organizations (eg, hospitals, labs) (Table 1).

**Table 1:** Attributes of Videos Included in Tier 1 Analysis

Category	n (%)
**Posted video**	
Organization	14 (16.9)
Individual	69 (83.1)
**Organization profit status**	
Nonprofit	0 (0)
For profit	14 (100)
**Organization type**	
Academic	0 (0)
Medical system/hospital/clinic	6 (42.9)
Society or professional organization	0 (0)
Medical laboratory	1 (7.1)
Other health care–related organization (eg, insurance company)^a^	5 (35.7)
Other non–health‐care‐related organization	2 (14.3)
**Individual type** ^b^	
Health care professional	15 (21.7)
Patient	56 (81.2)
**Type of video**	
Animated	1 (1.2)
Live action	74 (89.2)
Combination of animation and live action	3 (3.6)
Still photos	5 (6.0)

Abbreviation: NIPT, noninvasive prenatal testing.

a The “other” organization types include 5 for‐profit companies: an online health platform for individuals who are pregnant and newly parenting, an international prenatal health education platform, a company that provides pregnancy coaching and educational classes for individuals who are pregnant, an e‐book seller of pregnancy guides, and a company that provides prenatal and postpartum fitness classes that also produces a podcast.

b Two individuals who posted videos were both patient and a provider. Some individuals who posted were also doing so as a “for profit” company (eg, sonographer answering a question about the usefulness of NIPT from a viewer and details their personal story while also answering the question for the company video).

**Figure 2:**
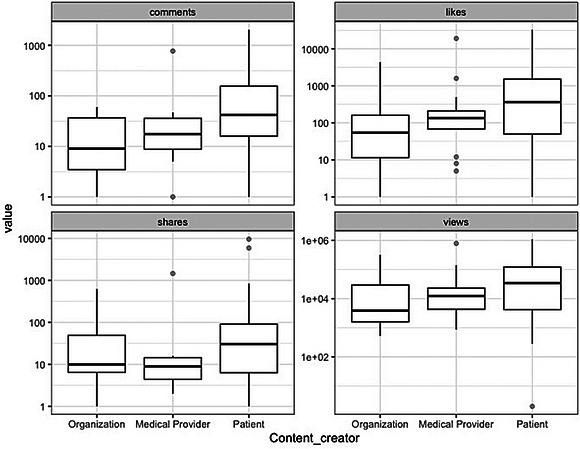
Log‐Scale Box Plots for Viewer Engagement With TikTok Videos

Figure 2 displays box plots of views, likes, shares, and comments to gauge engagement. Additional information, including means, SD, medians, ranges, and IQRs, is summarized in Supporting Information: Appendix S3. Video length varied widely (mean, 64.8 seconds; SD, 71.8; range, 6‐392 seconds), as did engagement. For instance, the mean overall views was 102,065.9 but ranged from 2 to more than 1 million. Patient/nonexpert videos consistently had higher engagement than those from providers or organizations.

### DISCERN Reliability and Quality of Information

The majority of TikTok videos were of poor or very poor quality, particularly patient videos, and had serious shortcomings in providing reliable sources of information for NIPT (Table 2). Within individual DISCERN items, only 8 videos were rated as balanced and unbiased, with medical professionals receiving the highest yes response rate (20%). Tables 2 and 3 summarize the DISCERN and bias results, respectively. See Supporting Information: Appendix S4 for the complete results of individual DISCERN items.

**Table 2:** DISCERN Overall Reliability and Quality of Information Score by Content Creator

	DISCERN Score n (%)				
Content Creator	Excellent (40‐45)	Good (34‐39)	Average (28‐33)	Poor (22‐27)	Very Poor (<22)
Organization	0 (0)	0 (0)	2 (14)	9 (64)	3 (21)
Health care professional	0 (0)	1 (7)	5 (33)	6 (40)	3 (20)
Patient	0 (0)	1 (2)	6 (11)	17 (30)	32 (57)

**Table 3:** Response to DISCERN Question “Is It Balanced and Unbiased” by Content Creator

	Not Balanced and Unbiased	Partially Balanced and Unbiased	Yes, Balanced and Unbiased
Content Creator Group^a^	n (%)	n (%)	n (%)
Organization	2 (14)	11 (79)	1 (7)
Health care professional	1 (7)	11 (73)	3 (20)
Patient	29 (52)	22 (39)	5 (9)

a Some videos qualified for more than one category.

Videos were further assessed for informational quality based on the presence of inaccurate information. The most common inaccuracy identified was conflating sex with gender, with 35 (42.2%) of videos using the term *gender* instead of *sex* when discussing chromosomal testing. This was particularly common in patient videos, with 52% conflating sex and gender (see Table 4). The second most common source of inaccuracy (31.3%) was content creators using language that suggests NIPT could determine the sex or gender of one's fetus (ie, deterministic). This deterministic inaccuracy was seen across organizations’, health care professionals’, and patients’ videos, with the lowest percentage for health care professionals (13%). Substantially fewer videos (8.4%) indicated that NIPT was diagnostic for other genetic or chromosomal conditions or presented other inaccurate information (4.8%). None of the health care professional videos presented either of these inaccuracies.

**Table 4:** Types of Inaccurate Information

	Conflating Sex With Gender	Screening Is Determinative of Sex or Gender	Screening Is Diagnostic for Genetic or Chromosomal Conditions	Other Cases of Inaccuracies
Content Creator Group^a^	n (%)	n (%)	n (%)	n (%)
Organization	4 (29)	5 (36)	4 (29)	0 (0)
Health care Professional	3 (20)	2 (13)	0 (0)	0 (0)
Patient	29 (52)	20 (36)	3 (5)	4 (7)
Total (% of total videos)	35 (42)	26 (31)	7 (8)	4 (5)

a Some videos qualified for more than one category.

### Conflation of Sex and Gender

Qualitative analysis revealed that many of the individual patient videos specifically focused on gender—for example, posting videos about their “gender reveal” and how the NIPT screening test was right or wrong in predicting the “gender” of the fetus. In comparison, both the health care provider and the organization videos were focused on providing empirical information about NIPT but would still frequently use the common cultural gendered language when the term sex would have been technically correct (20% of health care provider videos, 28.6% of organization videos), noting that the test can, for instance, tell “whether you're having a boy or a girl” (#73, 00:38) or mentioning that they have never seen NIPT “come back wrong gender wise” (#267, 00:26).

### Deterministic Framing

Many videos portrayed NIPT as confirmatory rather than predictive, particularly regarding fetal sex. By using language that suggests NIPT could determine the sex or gender of one's fetus or if the developing fetus has Down syndrome, they frame NIPT as deterministic. Patient videos often expressed frustration when NIPT results conflicted with later ultrasound findings: [on screen writing] “spent the last four months shopping for boy clothes just to find out the NIPT test was wrong and I'm having a girl” (#211, 00:01). Provider and organization videos were more likely to mention chromosomal conditions and clarify the screening nature of NIPT, although some still used deterministic language: “NIPT screens for any kind of chromosomal abnormalities, like Down syndrome, but also because it's a chromosomal analysis, it can figure out if you're having a boy or a girl” (#267, 00:01). Notably, it was common for the health care professionals’ videos to distinguish between NIPT as strictly a screening test and additional diagnostic tests that may be required later. The organizational videos were not as clear, with 3 organizational videos using deterministic language for both sex and other chromosomal abnormalities—for example, “it looks at your baby's chromosomes and can let you know if your baby has Down syndrome for example…you can also find out the gender if you want” (#196, 00:53).

### Intent and Tone

Video intent also influences perceived informational quality; assessment of health information quality is inherently tied to what communicators hope to convey.^34^ Patient videos often aimed to share personal experiences or fill information gaps, sometimes with humorous or skeptical tones. For example:

When I was 10 weeks pregnant we were really impatient and we wanted to know the gender of the baby. Now the NIPT test is just a screening tool. It's like 97% accurate in screening, but it does not diagnose and it can be wrong with the gender…but the two main reasons why I regret getting it is my insurance didn't cover it and our X chromosome for the female came back indeterminate…and it caused anxiety for no reason, according to my anatomy scan she's healthy (#269, 00:06).

Another common framing for patient videos was their shocked reaction to “wrong” information about sex provided by NIPT after an ultrasound revealed anatomy inconsistent with the sex they expected. The tone in these videos was sometimes negative and distrustful, whereas others presented their experience as humorous.

[Text on screen] We found out at our 20‐week anatomy scan that our ‘99.4% accurate’ NIPT blood test got the gender of our baby wrong! From week 14–20 we thought we were having a boy… Then at 21.5 weeks after 2 ultrasounds and a second blood test, we confirmed we're actually having a girl. [Spoken] Girl (#42, 00:01).

In comparison, both health care provider and organization videos typically had a neutral or positive tone. Provider videos clearly aimed to educate, using confident and professional presentation styles and typically wearing scrubs or displaying some other clear visual cue of their credentials, such as diplomas. Organizational videos often promoted services or products, sometimes even implying that their tests are better or different from what a patient may be offered elsewhere, such as letting viewers know that they can book “our new NIPT” test. A few videos from providers or organizations adopted a peer‐to‐peer format, presented as individuals sharing a personal story. In these cases, their professional affiliations were not disclosed in the video itself, and only an online search of the content creator revealed their association with an organization or a professional degree.

## DISCUSSION

This study assessed the quality of information about prenatal genetic screening on the social media platform TikTok, focusing on the description and contextualization of gender and sex terminology. Videos were posted by 3 distinct groups: patients/nonexperts, health care providers, and organizations. Although there were some generalities across videos, each of these content creator groups had unique styles. Patient videos often emphasized the “gender test” aspect of NIPT, conflated gender and sex, and portrayed NIPT as confirmatory. Health care provider videos aimed to educate, scored higher on DISCERN informational reliability, and presented themselves as trustworthy, although they also had the lowest engagement. Organization videos also sought to educate but frequently promoted services or products and a similar level of gender‐sex conflation as patient videos.

### Social Media as Education

ACOG recently updated its recommendations for patients with average or low‐risk pregnancies to allow providers to tailor their care, including both the possibility of fewer in‐person appointments and providing services in a different way. These new guidelines are intended to help patients access care.^1^ Yet, although these changes may reduce burdens for patients, they may also lead greater numbers of individuals who are pregnant to turn to social media, including TikTok, for information, guidance, or advice. Although TikTok recently added a new feature, Footnotes, which allows a select group of users to check accuracy, add context and background information, and provide links to additional resources,^35^ social media often lacks reliable health education. Videos designed to attract followers tend to prioritize engagement over accuracy,^36^ as seen in patient videos that teased future content or used colloquial language, reinforcing misconceptions, such as conflating gender and sex.

Perception also influences trust. For example, people are more likely to trust information presented confidently, regardless of source or veracity,^37^ and they are more likely to engage with a video presented by a “friend” or peer.^38^ Within EduTok (a campaign launched in 2019 by TikTok to label educational videos), researchers found that negatively framed and sensational videos had higher engagement,^36^ a trend mirrored in our findings, wherein patient videos had higher engagement and often included negative tones. Our findings regarding engagement align with those of similar studies. For instance, de Moel‐Mandel et al^39^ found that contraceptive information shared on TikTok by “hormonal coaches and health educators” obtained the fewest views, likes, and comments, whereas those shared by “general creators” were more likely to reject the use of hormonal contraception and express distrust of health care professionals in their videos. Additionally, in assessing videos addressing female pelvic floor conditions, Stephan et al^40^ revealed positive correlations between higher average misinformation scores and all measures of engagement (shares, likes, and overall engagement).

The structure of the US health care system is, in itself, potentially a barrier to effective health education. To resolve this, individuals, particularly those with lower health literacy or who distrust traditional care, may seek peer‐shared experiences online.

### Gender and Sex

Terms like “gender test” and celebratory phrases like “it's a boy!” reflect widespread societal conflation of sex and gender. Sex assigned at birth and a fetus's gender are traditionally conflated in colloquial settings, contributing to a binary understanding of both sex and gender. Even chromosomal sex, typically perceived as binary, is variable.^10^ Although these terms are distinct, they are frequently used interchangeably in public and professional contexts, with detailed discussions around the differences between sex and gender remaining fairly academic.^6^, ^7^ The result is a culturally complicated construct, making accurate communication challenging.

Although patient videos predictably referred to NIPT as a gender test, some health care professionals also reinforced this norm. Even videos that correctly described NIPT as a screening tool for chromosomal aneuploidies would sometimes use deterministic language when discussing fetal sex. When health care providers use the same inaccurate language as patients, they “reinforce an erroneous bioessentialist framework,” wherein “people with XX chromosomes or an apparent vulva are assigned female and socialized as girls, and people with XY chromosomes or an apparent penis are assigned male and socialized as boys.”^5^ ^, p.21^ They also perpetuate parental confusion and frustration when anatomy scans contradict earlier expectations because results surrounding issues of sex are not binary, and a fetus's genetic results about sex may not align with a child's eventual gender identity or performance.^41^, ^42^

Although it may take time for language use to change in social media, it is important for health care professionals to start the process of educating patients about the differences between sex and gender and the limitations of NIPT. This will involve a dedication to specificity in language in all patient interactions in which health care professionals use (and define) their terms related to sex chromosomes rather than mirroring inaccurate patient language or more culturally normative terms. If clinicians feel that this creates a disconnection with patients, they can note the terms that are used by patients—or that are more common colloquially—and then explain why they are using different terms. This is the case, whether in one‐on‐one clinical interactions or on social media platforms such as TikTok, wherein reference to a fetus's potential sex (rather than its gender) or genetic information about its sex chromosomes can offer a model for how lay communication about this topic can unfold as well.

Correspondingly, surveys of genetic counselors show that many patients prioritize fetal sex prediction when choosing NIPT and frequently assume that the primary, or sole, purpose of NIPT is fetal sex determination, yet most counselors lack protocols for addressing this motivation.^4^, ^43^ The creation of a training for counselors in specific language use related to sex chromosomes and sex prediction would go a long way toward alleviating this problem so that, ideally, patients begin to understand what NIPT results can tell them (the probability of fetal sex chromosomes, which may shed light on fetal sex) and what they cannot (fetal gender).

### Practice Implications

Patient‐shared experiences on TikTok offer valuable insights into the public understanding of NIPT. Inaccuracies in these videos highlight gaps in patient education and offer providers opportunities to address misunderstandings in clinical settings. Rather than dismissing social media, health care professionals should engage with it, both in the clinic and online, to improve the quality of health care information available to patients.

A handful of clinical recommendations to combat health inaccuracies online have been posited in recent years. These sources have a number of recommendations, including the following: (1) monitor information, both on social media and by asking patients what they are hearing online, to try to get ahead of what is out there; (2) address patient fears and help them to separate emotion from fact by asking open‐ended questions and listening carefully; (3) acknowledge actual risks of a given treatment or procedure; (4) when possible, cite sources and provide follow‐up resources; (5) provide materials from trusted sources; and (6) speak up beyond the clinic room by creating and sharing content on social media, writing an op‐ed, or participating in local education events.^44^, ^45^, ^46^ The American Psychological Association also recommends reporting inaccurate content and learning platform‐specific reporting mechanisms to have content removed if necessary.^47^

One subtle but effective strategy is to avoid mirroring, or using the same language as your client, when they use gendered language during NIPT discussions. Although mirroring builds rapport,^48^ Stevens et al found that most (84.4%) genetic counselors surveyed did not mirror gendered terminology in NIPT discussions, suggesting a passive educational approach that may help correct misconceptions.^43^ This same tactic could be useful in other clinical settings.

### Strengths, Limitations, and Future Directions

One key strength of this study is the diversity of our study team. We had an academically and generationally diverse team, enabling a multifaceted analysis of TikTok content. One limitation was the use of the DISCERN tool to quantitatively assess the informational quality of TikTok videos. DISCERN is widely used in video content analysis literature; however, it was originally designed to assess written health information, and we found it did not easily translate to video analysis, particularly the short personal attestation videos supplied via TikTok. An additional limitation is central to all research on social media. Any data pulled from social media companies use an application programming interface (API), including Apify, which this research team used. APIs act as intermediaries, but platforms can preprocess and shape data in ways that are unknown. In addition, TikTok and other social media video views are not random; the specific algorithms are kept secret but are known to prioritize content that gets people's attention. As a result, analyses are limited with regard to the platform's real‐world use.

Finally, future research should explore how often individuals who are of reproductive age rely on TikTok for prenatal and perinatal health information and how this influences their health care decisions, particularly when the information presented on social media is clearly narrative rather than didactic.

## CONCLUSION

The findings from this study underscore the influence of social media as a health care information source and reveal significant gaps in public understanding of NIPT, particularly regarding its use as a screening tool and limitations around sex or gender determination. Patient content creators have videos that receive the most engagement but tend to be more biased than those created by health care providers and organizations, in addition to more frequently conflating gender and sex and portraying NIPT as confirmatory. These results emphasize the importance of providers educating their patients with a particular focus on countering inaccurate information encountered on social media.

## CONFLICT OF INTEREST

The authors declare that they have no known competing financial interests or personal relationships that could have appeared to influence the work reported in this article.

## Supporting information




**Appendix S1**. Complete List of Data Elements Coded for in TikTok Video Analyses
**Appendix S2**. SRQR Checklist
**Appendix S3**. Video Engagement Table
**Appendix S4**. Results for the Complete List of Individual DISCERN Items

## References

[1] Screening for fetal chromosomal abnormalities . ACOG Practice Bulletin No. 226. Obstet Gynecol. 2020;136:e48‐e69. doi:10.1097/AOG.0000000000004084 32804883

[2] Salomon LJ , Sotiriadis A , Wulff CB , Odibo A , Akolekar R . Risk of miscarriage following amniocentesis or chorionic villus sampling: systematic review of literature and updated meta‐analysis. Ultrasound Obstet Gynecol. 2019;54(4):442‐451. doi:10.1002/uog.20353 31124209

[3] Bonner C , Trevena LJ , Gaissmaier W , et al. Current best practice for presenting probabilities in patient decision aids: fundamental principles. Med Decis Making. 2021;41(7):821‐833. doi:10.1177/0272989X21996328 33660551

[4] Agatisa PK , Mercer MB , Coleridge M , Farrell RM . Genetic counselors’ perspectives about cell‐free DNA: experiences, challenges, and expectations for obstetricians. J Genet Couns. 2018;27(6):1374‐1385. doi:10.1007/s10897-018-0268-y 29951719

[5] Llorin H , Lundeen T , Collins E , et al. Gender and sex inclusive approaches for discussing predicted fetal sex: a call for reflection and research. J Midwifery Womens Health. 2024;69(6):821‐825. doi:10.1111/jmwh.13663 39023042 PMC11622365

[6] Butler J . Bodies That Matter: On the Discursive Limits of Sex. Routledge; 1993.

[7] Butler J . Gender Trouble: Feminism and the Subversion of Identity. Routledge; 2006.

[8] Fausto‐Sterling A . Sexing the Body: Gender Politics and the Construction of Sexuality. Basic Books; 2000.

[9] McCartney M , Bewley S . Sex and gender should not be conflated in medical data. BMJ. 2025;389:r797. doi:10.1136/bmj.r797 40280625

[10] Jones T . Intersex studies: a systematic review of international health literature. SAGE Open. 2018;8(2):2158244017745577. doi:10.1177/2158244017745577

[11] World Health Organization, ed. Health Promotion Glossary of Terms . World Health Organization; 2021. Accessed June 26, 2025. https://www.who.int/publications/i/item/9789240038349

[12] Dyer J , Latendresse G , Cole E , Coleman J , Rothwell E . Content of first prenatal visits. Matern Child Health J. 2018;22(5):679‐684. doi:10.1007/s10995-018-2436-y 29335907 PMC5895499

[13] Légaré F , Ratté S , Gravel K , Graham ID . Barriers and facilitators to implementing shared decision‐making in clinical practice: update of a systematic review of health professionals' perceptions. Patient Educ Couns. 2008;73(3):526‐535. doi:10.1016/j.pec.2008.07.018 18752915

[14] Farrell RM , Pierce M , Collart C , et al. Making the most of the first prenatal visit: the challenge of expanding prenatal genetic testing options and limited clinical encounter time. Prenat Diagn. 2020;40(10):1265‐1271. doi:10.1002/pd.5752 32441820 PMC10114520

[15] McMullan M . Patients using the internet to obtain health information: how this affects the patient‐health professional relationship. Patient Educ Couns. 2006;63(1‐2):24‐28. doi:10.1016/j.pec.2005.10.006 16406474

[16] Wang X , Cohen RA . Health information technology use among adults: United States, July–December 2022: NCHS Data Brief no. 482. CDC National Center for Health Statistics. 2023. Accessed August 5, 2025. https://www.cdc.gov/nchs/products/databriefs/db482.htm

[17] Lim MSC , Molenaar A , Brennan L , Reid M , McCaffrey T . Young adults' use of different social media platforms for health information: insights from web‐based conversations. J Med Internet Res. 2022;24(1):e23656. doi:10.2196/23656 35040796 PMC8808344

[18] Kirkpatrick CE , Lawrie LL . TikTok as a source of health information and misinformation for young women in the United States: survey study. JMIR Infodemiology. 2024;4:e54663. doi:10.2196/54663 38772020 PMC11150891

[19] Gottfried J . Americans’ social media use. Pew Research Center. January 31, 2024. Accessed June 23, 2025. https://www.pewresearch.org/internet/2024/01/31/americans‐social‐media‐use/

[20] TikTok Scraper. Apify. 2022. Accessed January 5, 2025. https://apify.com/clockworks/tiktok‐scraper

[21] Charnock D , Shepperd S , Needham G , Gann R . DISCERN: an instrument for judging the quality of written consumer health information on treatment choices. J Epidemiol Community Health. 1999;53(2):105‐111. doi:10.1136/jech.53.2.105 10396471 PMC1756830

[22] Engin O , Songür K . Evaluation of YouTube videos as a source of information in traumatic brain injury rehabilitation: a cross‐sectional study. Medicine (Baltimore). 2024;103(32):e39254. doi:10.1097/md.0000000000039254 39121291 PMC11315507

[23] Coluk Y , Senocak MI . Patient education in the digital age: an analysis of quality and readability of online information on rhinoplasty. Medicine (Baltimore). 2024;103(32):e39229. doi:10.1097/md.0000000000039229 39121316 PMC11315473

[24] Yazla M , Akyon SH , Aybayar EA , et al. YouTube as a source of information in trauma management for ATLS (10th Edition) guidelines: evaluation of trauma management videos on YouTube. Emerg Med Int. 2024;1:7077469. doi:10.1155/2024/7077469 PMC11527541 39483788

[25] Benajiba N , Alhomidi M , Alsunaid F , et al. Video clips of the Mediterranean diet on YouTube (TM): a social media content analysis. Am J Health Promot. 2023;37(3):366‐374. doi:10.1177/08901171221132113 36191140 PMC9936443

[26] McBriar JD , Mishra A , Shah HA , Boockvar JA , Langer DJ , D'Amico RS . #Neurosurgery: a cross‐sectional analysis of neurosurgical content on TikTok. World Neurosurg X. 2023;17:100137. doi:10.1016/j.wnsx.2022.100137 36204176 PMC9529591

[27] Kim JH , Kim HK . Content and quality of YouTube regarding women's health: a scoping review. Korean J Women Health Nurs. 2023;29(3):179‐189. doi:10.4069/kjwhn.2023.08.19 37813661 PMC10565532

[28] Hsieh H‐F , Shannon SE . Three approaches to qualitative content analysis. Qual Health Res. 2005;15(9):1277‐1288. doi:10.1177/1049732305276687 16204405

[29] Braun V , Clarke V , Hayfield N , Terry G . Thematic analysis. In: Liamputtong P , ed. Handbook of Research Methods in Health Social Sciences. Springer Singapore; 2019:843‐860.

[30] Vears D , Gillam L . Inductive content analysis: a guide for beginning qualitative researchers. Focus Health Prof Educ. 2022;23(1):111‐127. doi:10.11157/fohpe.v23i1.544

[31] Lincoln YS , Guba EG . Naturalistic Inquiry. Sage Publications, Inc.; 1985.

[32] Morse JM . Critical analysis of strategies for determining rigor in qualitative inquiry. Qual Health Res. 2015;25(9):1212‐1222. doi:10.1177/1049732315588501 26184336

[33] O'Brien BC , Harris IB , Beckman TJ , Reed DA , Cook DA . Standards for reporting qualitative research: a synthesis of recommendations. Acad Med. 2014;89(9):1245‐1251. doi:10.1097/acm.0000000000000388 24979285

[34] Wu T , Deng Z , Zhang D , Buchanan PR , Zha D , Wang R . Seeking and using intention of health information from doctors in social media: the effect of doctor‐consumer interaction. Int J Med Informatics. 2018;115:106‐113. doi:10.1016/j.ijmedinf.2018.04.009 29779712

[35] Lindner E , Maheshwari S . TikTok asks users to help police misinformation. The New York Times. July 30, 2025. Accessed August 8, 2025. https://www.nytimes.com/2025/07/30/business/tiktok‐footnotes‐fact‐checking.html

[36] O'Donnell N , Jerin SI , Mu D . Using TikTok to educate, influence, or inspire? A content analysis of health‐related EduTok videos. J Health Commun. 2023;28(8):539‐551. doi:10.1080/10810730.2023.2234866 37434532

[37] Juteau A‐L , Holmes CJ , Brosseau‐Liard P . Confidence cues: epistemic or social? Cognitive Dev. 2025;74:101574. doi:10.1016/j.cogdev.2025.101574

[38] Hancock PA , Kessler TT , Kaplan AD , et al. How and why humans trust: a meta‐analysis and elaborated model. Front Psychol. 2023;14:1081086. doi:10.3389/fpsyg.2023.1081086 37051611 PMC10083508

[39] de Moel‐Mandel C , Donnelly A , Bugden M . “Do you know what birth control actually does to your body?”: assessing contraceptive information on TikTok. Perspect Sex Reprod Health. 2025;57(3):358‐367. doi:10.1111/psrh.70025 40589123 PMC12421087

[40] Stephan AP , Hauc SC , Marks VA , Bercik R , Rickey L . TikTok misinformation and user engagement in female pelvic floor conditions. Neurourol Urodyn. 2024;43(8):1956‐1961. doi:10.1002/nau.25519 38828831 PMC11496003

[41] Bahng J JA . Reframing the conversation around the “gender‐reveal”: how revealing fetal anatomy, not gender, is inclusive. OB GYN News. 2019;54(3):12.

[42] Browne TK . Why parents should not be told the sex of their fetus. J Med Ethics. 2017;43(1):5‐10. doi:10.1136/medethics-2015-102989 26846488

[43] Stevens C , Llorin H , Gabriel C , Mandigo C , Gochyyev P , Studwell C . Genetic counseling for fetal sex prediction by NIPT: challenges and opportunities. J Genet Couns. 2023;32(5):945‐956. doi:10.1002/jgc4.1703 37102371

[44] Anderer S . Patients are turning to TikTok for health information‐here's what clinicians need to know. JAMA. 2024;331(15):1262‐1264. doi:10.1001/jama.2024.1280 38517434

[45] Sharevski F , Loop JV , Jachim P , Devine A , Das S . ‘Debunk‐it‐yourself’: health professionals strategies for responding to misinformation on TikTok. Presented at: *Proceedings of the New Security Paradigms Workshop* ; 2025. doi:10.1145/3703465.3703469

[46] Davis C , Coles S . Confronting medical misinformation: tips from the trenches. Association of American Medical Colleges. August 11, 2022. Accessed August 8, 2025. https://www.aamc.org/news/confronting‐medical‐misinformation‐tips‐trenches

[47] Association AP . 8 recommendations for countering misinformation. American Psychological Association. March 1, 2024. Accessed July 1, 2025. https://www.apa.org/topics/journalism‐facts/misinformation‐recommendations

[48] Schuette JL , Yashar BM , Uhlmann WR . A Guide to Genetic Counseling. John Wiley & Sons; 2011.

