# Supplementary material for: The Patient, the Provider, and the TikTok Creator: Qualitative Analysis of the Content and Quality of Videos on Prenatal Genetic Screening
Source: J Midwifery Womens Health. 2026 Apr 13;71(4):618–27. doi: 10.1111/jmwh.70121 (PMC13460462; doi:10.1111/jmwh.70121)
Supplement: Supplementary file 1 [file JMWH-71-618-s001.docx]

**Supporting Information**

| **Appendix 1**  **Supplemental Table 1.** Complete list of data elements coded for in TikTok video analyses | | |
| --- | --- | --- |
| Video link | Date of retrieval | Exclusion criteria met |
| Whether the video was posted by an organization or individual | If an organization posted then profit status and type of organization | If an individual posted then whether it was a healthcare professional or a patient |
| Country of origin | Date posted | Number of days online |
| Length of video | Number of views | View rate (number of views/number of days since uploaded *100) |
| Number of likes | Number of shares | Number of comments |
| Whether the video was animated, live action, or combination | Whether the video contained LGBTQIA inclusive imagery and/or language | Whether the video mentioned sex chromosomes |
| mention of the fetus as boy or girl | mention of fetus in gender neutral terms | a brief description of the video |
| general observations (e.g., included bias, included preference for or against prenatal genetic screening) | tone of video (positive, neutral, or negative) | If it was a patient posting the video, did they indicate any distrust of healthcare professionals |
| If it was a patient posting the video, did they discuss the results of their prenatal genetic screening | Presentation of inaccuracy (conflating sex with gender, screening was described as determinative of sex or gender, screening was described as being diagnostic for chromosomal or genetic conditions, and other cases of inaccuracies) | Number of times inaccurate information was presented |

**Appendix 2.**

**SRQR Checklist**

|  | **Standards for Reporting Qualitative Research (SRQR)*** |  |
| --- | --- | --- |
|  | <http://www.equator-network.org/reporting-guidelines/srqr/> |  |
|  |  |  |
| **Title and abstract** | | **Page** |
|  | **Title** - Concise description of the nature and topic of the study Identifying the study as qualitative or indicating the approach (e.g., ethnography, grounded theory) or data collection methods (e.g., interview, focus group) is recommended | Title Page |
|  | **Abstract** - Summary of key elements of the study using the abstract format of the intended publication; typically includes background, purpose, methods, results, and conclusions | 1 |
|  |  |  |
| **Introduction** | |  |
|  | **Problem formulation** - Description and significance of the problem/phenomenon studied; review of relevant theory and empirical work; problem statement | 2-5 |
|  | **Purpose or research questio**n - Purpose of the study and specific objectives or questions | 5 |
|  |  |  |
| **Methods** | |  |
|  | **Qualitative approach and research paradigm** - Qualitative approach (e.g., ethnography, grounded theory, case study, phenomenology, narrative research) and guiding theory if appropriate; identifying the research paradigm (e.g., postpositivist, constructivist/ interpretivist) is also recommended; rationale** | 7 |
|  | **Researcher characteristics and reflexivity** - Researchers’ characteristics that may influence the research, including personal attributes, qualifications/experience, relationship with participants, assumptions, and/or presuppositions; potential or actual interaction between researchers’ characteristics and the research questions, approach, methods, results, and/or transferability | 7 |
|  | **Context** - Setting/site and salient contextual factors; rationale** |  |
|  | **Sampling strategy** - How and why research participants, documents, or events were selected; criteria for deciding when no further sampling was necessary (e.g., sampling saturation); rationale** | 5 |
|  | **Ethical issues pertaining to human subjects** - Documentation of approval by an appropriate ethics review board and participant consent, or explanation for lack thereof; other confidentiality and data security issues | 8 |
|  | **Data collection methods** - Types of data collected; details of data collection procedures including (as appropriate) start and stop dates of data collection and analysis, iterative process, triangulation of sources/methods, and modification of procedures in response to evolving study findings; rationale** | 5-7 |
|  | **Data collection instruments and technologies** - Description of instruments (e.g., interview guides, questionnaires) and devices (e.g., audio recorders) used for data collection; if/how the instrument(s) changed over the course of the study | 5-6, Supplement |
|  | **Units of study** - Number and relevant characteristics of participants, documents, or events included in the study; level of participation (could be reported in results) | 8, Supplement |
|  | **Data processing** - Methods for processing data prior to and during analysis, including transcription, data entry, data management and security, verification of data integrity, data coding, and anonymization/de-identification of excerpts | 6, Supplement |
|  | **Data analysis** - Process by which inferences, themes, etc., were identified and developed, including the researchers involved in data analysis; usually references a specific paradigm or approach; rationale** | 7 |
|  | **Techniques to enhance trustworthiness** - Techniques to enhance trustworthiness and credibility of data analysis (e.g., member checking, audit trail, triangulation); rationale** | 7 |
|  |  |  |
| **Results/findings** | |  |
|  | **Synthesis and interpretation** - Main findings (e.g., interpretations, inferences, and themes); might include development of a theory or model, or integration with prior research or theory | 8-11 |
|  | **Links to empirical data** - Evidence (e.g., quotes, field notes, text excerpts, photographs) to substantiate analytic findings | 9-11 |
|  |  |  |
| **Discussion** | |  |
|  | **Integration with prior work, implications, transferability, and contribution(s) to the field -** Short summary of main findings; explanation of how findings and conclusions connect to, support, elaborate on, or challenge conclusions of earlier scholarship; discussion of scope of application/generalizability; identification of unique contribution(s) to scholarship in a discipline or field | 11-15 |
|  | **Limitations** - Trustworthiness and limitations of findings | 15 |
|  |  |  |
| **Other** | |  |
|  | **Conflicts of interest** - Potential sources of influence or perceived influence on study conduct and conclusions; how these were managed | Author disclosures |
|  | **Funding** - Sources of funding and other support; role of funders in data collection, interpretation, and reporting | 16 |
|  |  |  |

**Appendix 3.**

| **Supplemental Table 2.** TikTok videos length of time and Engagement by content creator | | | | | | | |
| --- | --- | --- | --- | --- | --- | --- | --- |
|  | **Length of time (seconds)** | | | | | | |
|  | Mean | SD | Median | Max | Min | IQR (25%) | IQR (75%) |
| Organization | 40.21 | 27.52 | 33 | 90 | 8 | 20.75 | 55.25 |
| Medical provider | 59.67 | 72.61 | 45 | 304 | 10 | 19 | 59.5 |
| Patient | 75.68 | 83.51 | 55.5 | 392 | 6 | 14.75 | 106.25 |
|  | **Views** | | | | | | |
|  | Mean | SD | Median | Max | Min | IQR (25%) | IQR (75%) |
| Organization | 53209.43 | 96898.48 | 4191 | 318900 | 521 | 1599.5 | 30000 |
| Medical provider | 71621.07 | 200344.07 | 12400 | 785100 | 865 | 4458 | 23150 |
| Patient | 119023.91 | 234866.20 | 28550 | 1100000 | 2 | 4356.25 | 118925 |
|  | **Likes** | | | | | | |
|  | Mean | SD | Median | Max | Min | IQR (25%) | IQR (75%) |
| Organization | 441.21 | 1151.54 | 56 | 4350 | 1 | 11.5 | 160 |
| Medical provider | 1486.87 | 4860.80 | 134 | 19000 | 5 | 68 | 209 |
| Patient | 2197.36 | 5743.80 | 290.5 | 32700 | 1 | 51.25 | 1494 |
|  | **Shares** | | | | | | |
|  | Mean | SD | Median | Max | Min | IQR (25%) | IQR (75%) |
| Organization | 58.71 | 164.64 | 7 | 625 | 0 | 0.25 | 18.5 |
| Medical provider | 103.80 | 375.50 | 6 | 1461 | 0 | 2.5 | 13.5 |
| Patient | 333.82 | 1488.57 | 13 | 9586 | 0 | 0 | 41.25 |
|  | **Comments** | | | | | | |
|  | Mean | SD | Median | Max | Min | IQR (25%) | IQR (75%) |
| Organization | 10.86 | 19.33 | 0.5 | 60 | 0 | 0 | 8.25 |
| Medical provider | 65.67 | 195.16 | 14 | 769 | 0 | 3 | 27.5 |
| Patient | 173.07 | 376.70 | 38 | 2035 | 0 | 12.25 | 117.75 |

**Appendix 4**

| **Supplementary Table 3**. Number of videos meeting the noted responses for DISCERN individual item category | | | |
| --- | --- | --- | --- |
|  | **No**  **(# videos)** | **Partially Agree**  **(# videos)** | **Yes**  **(# videos)** |
| Are the aims clear? | 2 | 26 | 55 |
| Does it achieve its aims? | 9 | 41 | 31 |
| Is it relevant? | 25 | 47 | 11 |
| Is it clear what sources of information were used to compile the publication (other than the author or producer)? | 75 | 6 | 2 |
| Is it clear when the information used or reported in the publication was produced? | 77 | 4 | 1 |
| Is it balanced and unbiased? | 32 | 43 | 8 |
| Does it provide details of additional sources of support and information? | 74 | 6 | 3 |
| Does it refer to areas of uncertainty? | 41 | 27 | 15 |
| Does it describe how prenatal genetic screening works? | 63 | 17 | 3 |
| Does it describe the benefits of prenatal genetic screening? | 48 | 29 | 6 |
| Does it describe the risks of prenatal genetic screening? | 65 | 16 | 2 |
| Does it describe what would happen if nothing is done? | 73 | 7 | 3 |
| Does it describe how the choices affect overall quality of life? | 49 | 30 | 4 |
| Is it clear that there may be more than one possible choice? | 56 | 20 | 7 |
| Does it provide support for shared decision-making? | 68 | 8 | 7 |
